# NIHSS and inflammatory biomarkers predict 90-day poor outcome in older patients with acute ischemic stroke

**DOI:** 10.17305/bb.2026.14255

**Published:** 2026-05-15

**Authors:** Chunlan Hou, Qianwei Zheng, Zilong Yang, Baoai Wang

**Affiliations:** 1 Fenyang College, Shanxi Medical University, Fenyang, China; 2 Department of Neurology, Fenyang Hospital of Shanxi Province, Fenyang, China; 3 Department of Neurology, First Hospital of Shanxi Medical University, Taiyuan, China

**Keywords:** Acute ischemic stroke, interleukin-17A, matrix metalloproteinase-9, prostaglandin-endoperoxide synthase 2, prognosis

## Abstract

Early prediction of functional outcomes following acute ischemic stroke (AIS) in older adults presents significant clinical challenges. Inflammation-related biomarkers may enhance clinical severity scores for early risk stratification. This single-center prospective observational study aimed to develop and internally validate an exploratory prediction model that integrates the admission National Institutes of Health Stroke Scale (NIHSS) score with serum inflammatory biomarkers to predict 90-day poor outcomes in older patients with AIS. Consecutive patients aged 60 years and older, with magnetic resonance imaging-confirmed AIS and blood sampling within 24 hours of stroke onset, were enrolled from May to December 2025. Patients with a history of previous strokes, active infections or autoimmune diseases, severe hepatic or renal dysfunction, incomplete data, or those who received intravenous thrombolysis or endovascular treatment were excluded from the study. Poor outcomes were defined as a modified Rankin Scale (mRS) score of 3 or higher at 90 days. Serum levels of prostaglandin-endoperoxide synthase 2 (PTGS2), matrix metalloproteinase-9 (MMP-9), and interleukin-17A (IL-17A) were measured using enzyme-linked immunosorbent assay (ELISA). Among the 171 patients included in the final analysis, 50 experienced poor outcomes, while 121 had favorable outcomes. In a multivariable logistic regression analysis adjusted for prespecified clinical covariates, admission NIHSS score, MMP-9, PTGS2, and IL-17A were found to be independently associated with poor outcomes and were retained in a simplified nomogram. The combined model demonstrated an apparent area under the receiver operating characteristic curve (AUC) of 0.907 (95% confidence interval [CI], 0.864–0.950) and a bootstrap-corrected concordance index (C-index) of 0.897. Calibration was acceptable, with a bootstrap-corrected calibration intercept of 0.0004 and a slope of 0.916. Additionally, decision curve analysis (DCA) indicated a potential net benefit across clinically plausible threshold probabilities. However, the model development and evaluation were conducted within a modest, selected, single-center cohort, and the number of poor outcome events was limited. Furthermore, the routine availability of inflammatory biomarker testing is lacking in many clinical settings, and the exclusion of reperfusion-treated patients restricts the model’s generalizability. Consequently, this NIHSS- and biomarker-based model should be regarded as exploratory and hypothesis-generating. External validation in larger, multicenter, and more heterogeneous cohorts is essential before clinical application.

## Introduction

Acute ischemic stroke (AIS) is a leading cause of death and disability among older adults [1]. As the global population ages, the incidence of AIS among older patients continues to rise, placing a significant burden on public health systems [2]. AIS is a heterogeneous condition that includes various etiological and pathophysiological subtypes, such as large-artery atherosclerotic stroke, cardioembolic stroke, small-vessel occlusion or lacunar infarction, stroke of determined etiology, and stroke of undetermined etiology. Accurate differentiation of these subtypes is crucial in clinical studies and therapeutic trials, as they exhibit distinct risk-factor profiles, baseline stroke severity, therapeutic strategies, inflammatory characteristics, and functional outcomes [3]. Therefore, the heterogeneity of stroke subtypes must be considered when developing and interpreting prognostic models for AIS, particularly in older patients who frequently present with multiple comorbidities and overlapping vascular mechanisms. Compared to younger patients, older individuals with AIS typically have a higher burden of comorbidities, increased likelihood of frailty, and diminished neurological reserve. Consequently, they are more prone to severe neurological deficits, a greater incidence of in-hospital complications, and poorer functional recovery, resulting in increased variability in clinical outcomes [4, 5]. Thus, creating precise prognostic tools tailored to the older population is of paramount clinical importance for early risk stratification and individualized intervention.

Currently, most prognostic models widely utilized in clinical practice for stroke were developed in mixed-age populations, necessitating further validation of their applicability and predictive performance in older subgroups [6]. Additionally, inflammatory mechanisms pertinent to stroke prognosis in older adults have often been inadequately incorporated into existing models [7]. Recent studies have increasingly demonstrated that inflammatory responses play a critical role in the progression and repair of ischemic brain injury [8–10]. Among these factors, matrix metalloproteinase-9 (MMP-9) can compromise blood-brain barrier integrity, exacerbating cerebral edema and secondary neuronal injury [11]. Interleukin-17A (IL-17A), an effector cytokine in the T helper 17 (Th17)-related inflammatory pathway, can enhance local inflammatory cascades and promote neuronal apoptosis [12]. Prostaglandin-endoperoxide synthase 2 (PTGS2) is a key inducible enzyme in the arachidonic acid-prostaglandin pathway, playing a significant role in post-ischemic inflammatory responses and oxidative stress following ischemic brain injury [13, 14].

These three biomarkers are involved in blood-brain barrier disruption, amplification of immune-inflammatory responses, and prostaglandin-related inflammatory pathways, potentially reflecting the injury process after AIS in older adults from various pathophysiological perspectives. However, studies that jointly evaluate their prognostic value specifically within the older population are limited. Consequently, this study enrolled patients aged 60 years and older with AIS, integrating routine clinical indicators with serum levels of PTGS2, MMP-9, and IL-17A, identifying independent predictors, and constructing a nomogram prediction model. The objective was to ascertain whether the integration of clinical severity and inflammatory biomarkers could enhance early risk stratification for poor 90-day outcomes in older patients with AIS.

## Materials and methods

### Study population

This single-center prospective observational study enrolled older patients with AIS (aged ≥ 60 years) who were hospitalized at Fenyang Hospital of Shanxi Province between May 2025 and December 2025, with their clinical data collected. The inclusion criteria were: (1) diagnosis of AIS according to the 2023 updated guidelines of the Chinese Stroke Association [15], confirmed by brain magnetic resonance imaging (MRI); (2) onset-to-blood sampling time of less than 24 h; (3) age of at least 60 years. The exclusion criteria included: (1) a history of previous stroke; (2) concomitant intracranial hemorrhage, tumor, active infection, or autoimmune disease; (3) severe hepatic or renal dysfunction (Child-Pugh class C or estimated glomerular filtration rate (eGFR) <30 mL/min/1.73 m^2^); (4) receipt of intravenous thrombolysis or endovascular treatment; and (5) incomplete clinical data. Patients who did not complete the 90-day follow-up or had unavailable 90-day outcome data were excluded from the final analysis.

### Clinical assessment and outcome evaluation

Baseline neurological severity was assessed using the National Institutes of Health Stroke Scale (NIHSS) upon admission [16] by trained neurologists experienced in standardized stroke assessment. The NIHSS score analyzed was the first recorded score after admission and prior to treatment-related or follow-up outcome assessments. Demographic characteristics, vascular risk factors, medical history, and baseline clinical data were collected from the electronic medical record system using a standardized case report form.

MRI was reviewed to confirm the diagnosis of AIS and characterize infarct features. Infarct location was categorized based on the involved vascular territory or anatomical region, such as anterior or posterior circulation, according to the identified acute ischemic lesion on diffusion-weighted imaging. Infarct size was classified on diffusion-weighted imaging according to the maximum diameter and anatomical extent of the symptomatic lesion: large infarction was defined as involving more than one cerebral lobe or having a maximum diameter greater than 5.0 cm; medium infarction as involving less than one cerebral lobe with a maximum diameter between 3 cm and 5 cm; small infarction as a lesion with a maximum diameter between 1.5 cm and 3 cm; and lacunar infarction as a lesion with a maximum diameter of 1.5 cm or less.

Stroke etiology was classified according to the Trial of Org 10172 in Acute Stroke Treatment (TOAST) criteria [17], which include large-artery atherosclerosis, cardioembolism, small-vessel occlusion, stroke of other determined etiology, and stroke of undetermined etiology. TOAST subtype classification was independently determined by two trained neurologists based on clinical presentation, neuroimaging findings, vascular imaging, cardiac evaluation, and relevant laboratory results. Disagreements were resolved through discussion with a senior stroke neurologist.

Carotid plaque was evaluated using carotid ultrasonography during hospitalization and was defined as a focal structure encroaching into the arterial lumen by at least 0.5 mm, or 50% of the surrounding intima-media thickness, or having a thickness greater than 1.5 mm measured from the media-adventitia interface to the intima-lumen interface.

The primary endpoint was the 90-day functional outcome following stroke onset, defined according to the modified Rankin Scale (mRS) [18]. A poor outcome was classified as an mRS score of 3 or higher, while a favorable outcome was classified as an mRS score of 0–2. The 90-day mRS was obtained through structured telephone interviews conducted by trained investigators. If the patient was unable to respond reliably, information was gathered from a caregiver or legally authorized representative. All outcome assessors were blinded to the laboratory test results obtained at enrollment. Patients lacking available 90-day mRS data were excluded from the final analysis.

### Serum sample collection

For all older patients with AIS, 5 mL of venous blood was collected within 24 h of stroke onset. Blood samples were allowed to stand at room temperature for 30 min before being centrifuged at 3000 rpm for 15 min at 4^∘^C. The serum was then separated, aliquoted, and stored at –80 ^∘^C to avoid repeated freeze-thaw cycles. Serum concentrations of PTGS2, MMP-9, and IL-17A were measured using enzyme-linked immunosorbent assay (ELISA). Measurements were conducted using commercially available ELISA kits according to the manufacturers’ instructions. The assay characteristics were as follows: for MMP-9 (Ruixin Biotech, catalog no. RX105770H), the analytical range was 1.5–48 ng/mL, the sensitivity was <0.1 ng/mL, the manufacturer-reported intra-assay coefficient of variation (CV) was <10%, and the inter-assay CV was <15%; for PTGS2 (Ruixin Biotech, catalog no. RX2D182956), the analytical range was 20–640 pg/mL, the sensitivity was <1.0 pg/mL, the intra-assay CV was <10%, and the inter-assay CV was <15%; and for IL-17A (Ruixin Biotech, catalog no. RX106159H), the analytical range was 1–32 pg/mL, the sensitivity was <0.1 pg/mL, the intra-assay CV was <10%, and the inter-assay CV was <15%. All samples were measured in duplicate, and the mean value of duplicate measurements was used for analysis. The assay range and CVs were within the limits specified by the manufacturer. Samples exhibiting visible hemolysis, lipemia, insufficient volume, or duplicate measurements with excessive variability were re-assayed following predefined laboratory quality-control procedures. Laboratory personnel were blinded to the clinical grouping information.

### Model construction and evaluation

The poor 90-day outcome was designated as the dependent variable. The candidate variables considered in the analysis included baseline demographic and clinical characteristics (age, sex, smoking status, alcohol consumption, history of diabetes mellitus, hypertension, coronary heart disease, and atrial fibrillation), infarct-related variables (infarct size grade, TOAST classification, infarct location, and carotid plaque), onset-to-blood sampling time, laboratory variables (triglycerides, total cholesterol, low-density lipoprotein cholesterol, high-density lipoprotein cholesterol, homocysteine, fasting blood glucose, glycated hemoglobin, and D-dimer), admission NIHSS score, and three serum biomarkers (MMP-9, PTGS2, and IL-17A).

Univariate logistic regression was initially conducted to assess the association between each variable and poor outcome. Based on clinical relevance, prior evidence, and the limited number of outcome events, age, sex, history of hypertension, history of diabetes mellitus, history of coronary heart disease, and onset-to-blood sampling time were prespecified as adjustment variables. The admission NIHSS score and the three serum biomarkers (MMP-9, PTGS2, and IL-17A) were subsequently incorporated into a multivariable logistic regression model alongside these prespecified covariates to identify independent predictors. While the final simplified prediction model retained only the predictors that remained independently associated with poor outcomes, we acknowledge that this preceding adjustment step may have contributed to a degree of selection-related optimism.

Given that there were 50 poor outcome events in this cohort, and to mitigate the risk of overfitting, only the predictors that remained independently associated with poor outcomes were included in the final simplified prediction model. This model comprised four predictor parameters (admission NIHSS score, MMP-9, PTGS2, and IL-17A), resulting in an events-per-variable ratio of 12.5 (50/4). To enhance the interpretability and comparability of effect estimates across biomarkers measured on different scales, we standardized MMP-9, PTGS2, and IL-17A as z-scores and reported odds ratios (ORs) per 1 standard deviation (SD) increase. The original measurement scales were preserved in the final prediction model and nomogram to facilitate direct estimation of individual risk.

Variance inflation factors were employed to assess multicollinearity, and restricted cubic splines were utilized to evaluate the linearity of associations between continuous variables and the outcome. Model performance was assessed using receiver operating characteristic (ROC) curves, the concordance index (C-index), 1,000-bootstrap resampling, calibration plots, calibration intercept, calibration slope, the Hosmer–Lemeshow test, and decision curve analysis (DCA). Internal validation was performed using 1,000 bootstrap resamples with replacement from the final analytical cohort. In each bootstrap resample, the model was refitted and tested to estimate the optimism in model performance. Bootstrap-corrected discrimination and calibration estimates were calculated to evaluate internal stability, which did not constitute external validation. Calibration intercept and slope were used to quantify systematic miscalibration and overfitting, with ideal values of 0 and 1, respectively. Because apparent calibration measures may be optimistic when evaluated in the development dataset, bootstrap-corrected calibration intercept and slope estimates were also obtained. Given that multiple ROC analyses and pairwise DeLong comparisons were conducted, the resulting *P* values should be interpreted cautiously due to an increased risk of type I error. These comparisons were exploratory, and greater emphasis was placed on the magnitude of differences in the area under the ROC curve (AUC) rather than on nominal statistical significance alone. All tests were two-sided, and a *P* value <0.05 was deemed statistically significant.

**Figure 1.**
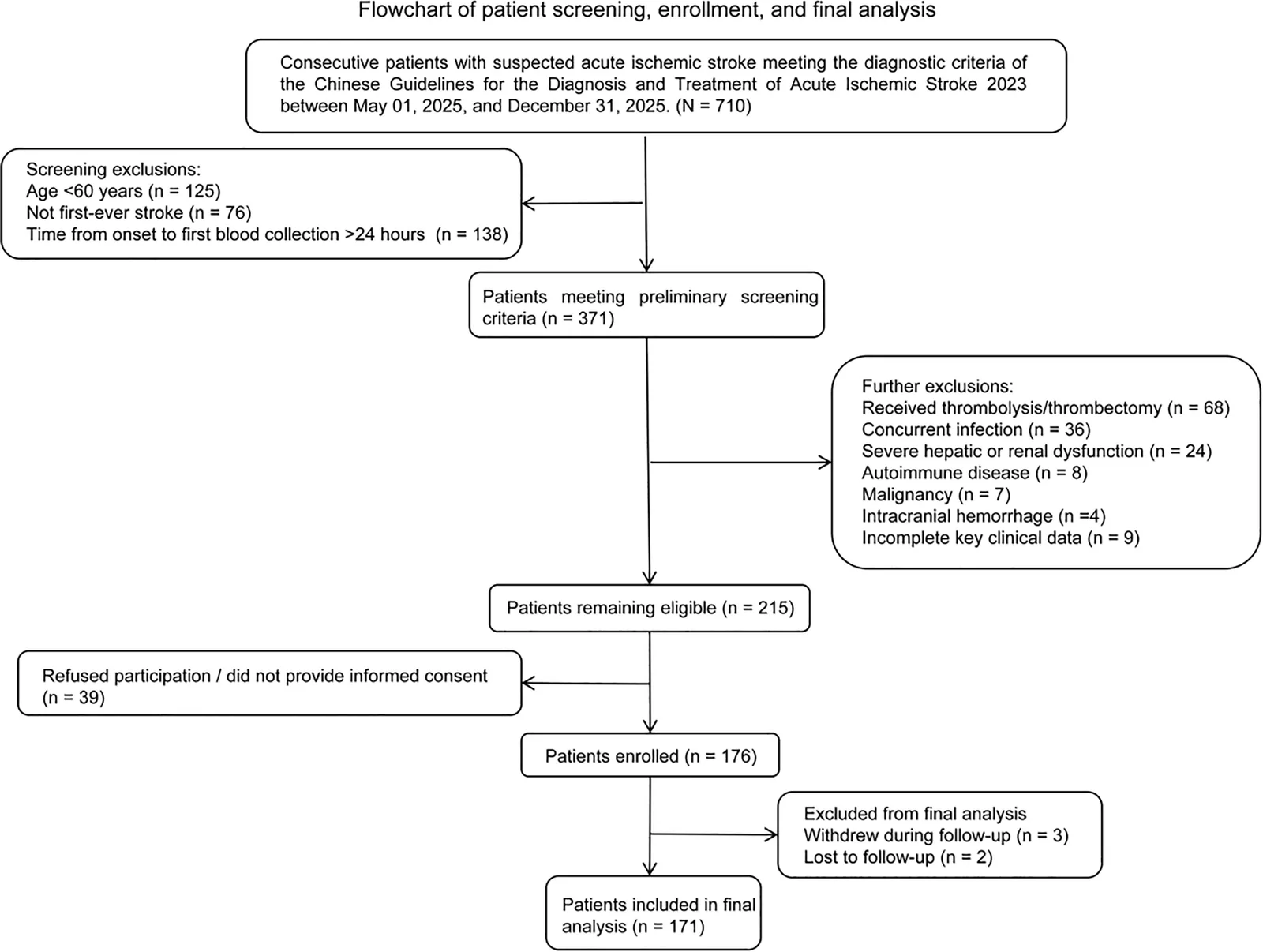
**Flowchart of patient screening, enrollment, and final analysis.** During the study period, 710 consecutive patients with acute ischemic stroke were screened. Following screening exclusions and additional eligibility assessments, 215 patients were deemed eligible. Of these, 39 patients either declined participation or did not provide informed consent, resulting in 176 enrolled patients. Five patients were excluded from the final analysis due to unavailability of 90-day outcome data, leading to a final cohort of 171 patients included in the analysis.

### Ethical statement

This study received approval from the Ethics Committee of Fenyang Hospital in Shanxi Province (Approval No. 2025061). All procedures involving human participants and the collection and use of human blood samples were conducted in accordance with relevant guidelines, regulations, and the Declaration of Helsinki and its subsequent amendments. Written informed consent was obtained from all participants or their legally authorized representatives.

### Statistical analysis

Statistical analyses were conducted using R software version 4.4.0. Continuous variables were first evaluated for normality using the Shapiro-Wilk test. Normally distributed data are presented as mean ± SD, with comparisons between groups performed using the independent-samples *t*-test. Non-normally distributed data are presented as median (P25, P75), with group comparisons executed using the Mann-Whitney *U* test. Categorical variables are presented as n (%) and were compared using the chi-square test or Fisher’s exact test, as appropriate. All variables included in the analysis were complete, with no missing data.

## Results

### Flowchart of study participant inclusion

The flowchart depicting participant screening is presented in Figure 1. A total of 710 consecutive patients with AIS were screened during the study period. After applying exclusion criteria and conducting further eligibility assessments, 215 patients remained eligible. Among these, 39 patients declined participation or did not provide informed consent, resulting in 176 enrolled patients. Five patients were excluded due to incomplete follow-up data, leading to a final analysis cohort of 171 patients. Of these, 121 patients exhibited a favorable outcome, while 50 patients experienced a poor outcome.

### Baseline characteristics of older patients with AIS

Baseline comparisons revealed no statistically significant differences between the favorable outcome group and the poor outcome group regarding age, sex, smoking history, alcohol consumption, history of diabetes mellitus, history of hypertension, history of coronary heart disease, history of atrial fibrillation, infarct size grade, onset-to-blood sampling time, blood lipid profiles, homocysteine, glucose-related indicators, D-dimer, TOAST classification, infarct location, or carotid plaque status (all *P* > 0.05). In contrast, the poor outcome group exhibited significantly higher admission NIHSS scores and elevated serum levels of MMP-9, PTGS2, and IL-17A (all *P* < 0.001). Detailed clinical characteristics are presented in Table 1.

**Table 1:** Comparison of baseline characteristics between the favorable outcome group and the poor outcome group

**Variables**	**Favorable outcome (*n* ═ 121)**	**Poor outcome (*n* ═ 50)**	**Statistic**	***P* value**
Age, years	67.00 (62.00, 69.00)	68.00 (67.00, 70.00)	Z ═ –1.62	0.106
Sex			χ^2^ ═ 1.42	0.233
Female	48 (39.67%)	15 (30.00%)		
Male	73 (60.33%)	35 (70.00%)		
Smoking			χ^2^ ═ 3.13	0.077
Yes	34 (28.10%)	21 (42.00%)		
No	87 (71.90%)	29 (58.00%)		
Alcohol consumption			χ^2^ ═ 3.00	0.083
Yes	16 (13.22%)	12 (24.00%)		
No	105 (86.78%)	38 (76.00%)		
Diabetes mellitus			χ^2^ ═ 0.16	0.690
Yes	28 (23.14%)	13 (26.00%)		
No	93 (76.86%)	37 (74.00%)		
Hypertension			χ^2^ ═ 1.10	0.293
Yes	77 (63.64%)	36 (72.00%)		
No	44 (36.36%)	14 (28.00%)		
Coronary heart disease			-	0.083
Yes	5 (4.13%)	6 (12.00%)		
No	116 (95.87%)	44 (88.00%)		
Atrial fibrillation			χ^2^ ═ 1.71	0.191
Yes	11 (9.09%)	8 (16.00%)		
No	110 (90.91%)	42 (84.00%)		
Infarct size grade			-	0.281
≤1.5 cm	73 (60.33%)	25 (50.00%)		
>1.5 cm and ≤3 cm	30 (24.79%)	14 (28.00%)		
>3 cm and ≤5 cm	18 (14.88%)	10 (20.00%)		
>5.0 cm	0 (0.00%)	1 (2.00%)		
Onset-to-blood sampling time, h	9.00 (6.00, 15.00)	11.00 (7.00, 13.75)	Z ═ –0.71	0.481
TG, mmol/L	1.19 (0.92, 1.92)	1.14 (0.89, 1.60)	Z = 0.82	0.414
TC, mmol/L	3.95 (3.55, 4.39)	4.04 (3.29, 4.78)	Z ═ –0.77	0.443
LDL-C, mmol/L	2.62 (1.98, 3.10)	2.58 (2.13, 3.05)	Z = 0.09	0.926
HDL-C, mmol/L	1.01 (0.88, 1.22)	1.00 (0.80, 1.25)	Z = 0.26	0.792
Homocysteine, µmol/L	15.80 (10.90, 22.00)	18.20 (13.48, 23.00)	Z ═ –1.48	0.138
Fasting blood glucose, mmol/L	5.16 (4.63, 5.97)	5.10 (4.61, 5.90)	Z = 0.10	0.920
Glycated hemoglobin, %	5.70 (5.40, 6.30)	5.60 (5.33, 6.20)	Z = 0.69	0.490
D-dimer, mg/L	0.50 (0.40, 0.80)	0.50 (0.42, 0.69)	Z = 0.33	0.738
TOAST classification			-	0.203
Large-artery atherosclerosis	50 (41.32%)	21 (42.00%)		
Cardioembolic subtype	10 (8.26%)	7 (14.00%)		
Small-artery occlusion	59 (48.76%)	19 (38.00%)		
Other determined or undetermined etiology	2 (1.65%)	3 (6.00%)		
Infarct location			χ^2^ ═ 0.71	0.399
Anterior circulation infarction	69 (57.03%)	32 (64.00%)		
Posterior circulation infarction	52 (42.97%)	18 (36.00%)		
Carotid plaque			χ^2^ ═ 3.57	0.059
Yes	71 (58.68%)	37 (74.00%)		
No	50 (41.32%)	13 (26.00%)		
Admission NIHSS score, points	2.00 (1.00, 4.00)	4.00 (3.00, 6.00)	Z ═ –5.23	<0.001
MMP-9, ng/mL	9.21 (7.80, 10.82)	11.14 (9.73, 12.37)	Z ═ –4.24	<0.001
PTGS2, pg/mL	106.81 (88.82, 129.86)	142.05 (125.15, 162.45)	Z ═ –6.38	<0.001
IL-17A, pg/mL	5.42 (4.59, 6.52)	6.76 (6.17, 7.26)	Z ═ –5.59	<0.001

Continuous variables are presented as median (P25, P75), and group comparisons were conducted using the Mann–Whitney *U* test. Categorical variables are reported as n (%), with group comparisons performed using the Pearson χ^2^ test or Fisher’s exact test, denoted by “–” when Fisher’s exact test was applied. **Abbreviations:** HDL-C, high-density lipoprotein cholesterol; IL-17A, interleukin-17A; LDL-C, low-density lipoprotein cholesterol; MMP-9, matrix metalloproteinase-9; NIHSS, National Institutes of Health Stroke Scale; P25, 25th percentile; P75, 75th percentile; PTGS2, prostaglandin-endoperoxide synthase 2; TC, total cholesterol; TG, triglycerides; TOAST, Trial of Org 10172 in Acute Stroke Treatment.

### Univariate logistic regression analysis

To quantify the strength of association between each variable and poor 90-day outcome, a univariate logistic regression analysis was performed (Table 2). The results indicated that admission NIHSS score, MMP-9, PTGS2, and IL-17A were all significantly associated with poor outcomes. A higher admission NIHSS score correlated with an increased risk of poor outcome (OR = 1.64, 95% confidence interval [CI]: 1.34–2.05, *P* < 0.001). Elevated MMP-9 levels were also linked to an increased risk of poor outcome (OR = 1.42, 95% CI: 1.21–1.70, *P* < 0.001). Increased PTGS2 levels were associated with a higher risk of poor outcome (OR = 1.05, 95% CI: 1.03–1.07, *P* < 0.001). Similarly, elevated IL-17A was significantly correlated with an increased risk of poor outcome (OR = 2.14, 95% CI: 1.62–2.95, *P* < 0.001). Additionally, smoking history, alcohol consumption, history of coronary heart disease, and carotid plaque demonstrated associations with poor outcomes that approached statistical significance, although they did not reach the threshold for significance. Some categorical subgroups contained very few patients, and the corresponding regression estimates should be interpreted cautiously as exploratory.

**Table 2:** Univariate logistic regression analysis of factors associated with adverse outcomes in older patients with AIS

**Variables**	**Level**	**β**	**SE**	**Wald Z**	**OR (95% CI)**	***P* value**
Age	Per 1-unit increase	0.03	0.03	0.94	1.03 (0.97, 1.09)	0.347
Sex	Male	0.43	0.36	1.19	1.53 (0.77, 3.17)	0.235
Smoking	Yes	0.62	0.35	1.76	1.85 (0.93, 3.69)	0.079
Alcohol consumption	Yes	0.73	0.43	1.71	2.07 (0.89, 4.77)	0.087
Diabetes mellitus	Yes	0.15	0.39	0.40	1.17 (0.53, 2.46)	0.691
Hypertension	Yes	0.39	0.37	1.05	1.47 (0.73, 3.09)	0.295
Coronary heart disease	Yes	1.15	0.63	1.83	3.16 (0.91, 11.48)	0.068
Atrial fibrillation	Yes	0.64	0.50	1.29	1.91 (0.69, 5.04)	0.196
Infarct size grade	>1.5 cm and ≤3 cm	0.31	0.40	0.78	1.36 (0.62, 2.96)	0.437
	>3 cm and ≤5 cm	0.48	0.46	1.06	1.62 (0.65, 3.94)	0.290
	>5.0 cm	-	-	-	-	-
Onset-to-blood sampling time	Per 1-unit increase	0.02	0.03	0.63	1.02 (0.96, 1.09)	0.530
TG	Per 1-unit increase	–0.21	0.18	–1.13	0.81 (0.54, 1.12)	0.258
TC	Per 1-unit increase	0.26	0.19	1.36	1.30 (0.89, 1.90)	0.175
LDL-C	Per 1-unit increase	0.11	0.20	0.54	1.12 (0.75, 1.66)	0.589
HDL-C	Per 1-unit increase	–0.17	0.31	–0.53	0.85 (0.32, 1.17)	0.595
Homocysteine	Per 1-unit increase	0.01	0.01	0.68	1.01 (0.99, 1.03)	0.498
Fasting blood glucose	Per 1-unit increase	–0.01	0.09	–0.14	0.99 (0.82, 1.16)	0.889
Glycated hemoglobin	Per 1-unit increase	–0.12	0.14	–0.83	0.89 (0.67, 1.15)	0.406
D-dimer	Per 1-unit increase	–0.17	0.26	–0.66	0.85 (0.46, 1.31)	0.510
TOAST classification	Cardioembolic subtype	0.51	0.56	0.92	1.67 (0.54, 4.95)	0.359
	Small-artery occlusion	–0.27	0.37	–0.72	0.77 (0.37, 1.59)	0.473
	Other determined or undetermined etiology	1.27	0.95	1.34	3.57 (0.55, 28.63)	0.180
Infarct location	Anterior circulation infarction	0.29	0.35	0.84	1.34 (0.68, 2.68)	0.399
Carotid plaque	Yes	0.70	0.37	1.87	2.00 (0.99, 4.27)	0.061
Admission NIHSS score	Per 1-unit increase	0.49	0.11	4.53	1.64 (1.34, 2.05)	<0.001
MMP-9	Per 1-unit increase	0.35	0.09	4.09	1.42 (1.21, 1.70)	<0.001
PTGS2	Per 1-unit increase	0.05	0.01	5.69	1.05 (1.03, 1.07)	<0.001
IL-17A	Per 1-unit increase	0.76	0.15	5.02	2.14 (1.62, 2.95)	<0.001

Univariate logistic regression analysis was employed to evaluate the associations between clinical characteristics, laboratory indicators, and poor outcomes. Results are expressed as ORs with 95% CIs. For categorical variables, the OR indicates the odds relative to the reference group; for continuous variables, the OR reflects the change in the risk of poor outcomes associated with each one-unit increase in the respective indicator. Caution is advised when interpreting estimates for categories with sparse data, as these may be unstable and should be considered exploratory. Due to insufficient data, the regression coefficient and associated statistics for the infarct-size category “>5.0 cm” could not be reliably estimated and are therefore excluded. A *P* value of less than 0.05 was deemed statistically significant. **Abbreviations:** AIS, acute ischemic stroke; CI, confidence interval; HDL-C, high-density lipoprotein cholesterol; IL-17A, interleukin-17A; LDL-C, low-density lipoprotein cholesterol; MMP-9, matrix metalloproteinase-9; NIHSS, National Institutes of Health Stroke Scale; OR, odds ratio; PTGS2, prostaglandin-endoperoxide synthase 2; SE, standard error; TC, total cholesterol; TG, triglycerides; TOAST, Trial of Org 10172 in Acute Stroke Treatment; β, beta coefficient.

### Identification of independent predictors

In the multivariable logistic regression model adjusted for the prespecified covariates of age, sex, history of hypertension, history of diabetes mellitus, history of coronary heart disease, and onset-to-blood sampling time (Table 3), admission NIHSS score (OR = 1.77, 95% CI: 1.34–2.46, *P* < 0.001), MMP-9 (OR = 1.33, 95% CI: 1.06–1.72, *P* ═ 0.020), PTGS2 (OR = 1.04, 95% CI: 1.02–1.06, *P* < 0.001), and IL-17A (OR = 1.79, 95% CI: 1.22–2.74, *P* ═ 0.005) remained independently associated with poor 90-day outcomes, indicating that these four indicators could be integrated as candidate variables in the final prediction model. To facilitate interpretation, we also analyzed the three biomarkers on a standardized scale. The associations remained directionally consistent, with higher levels of MMP-9, PTGS2, and IL-17A each correlating with increased odds of poor 90-day outcome per 1 SD increase (Table S1).

**Figure 2.**
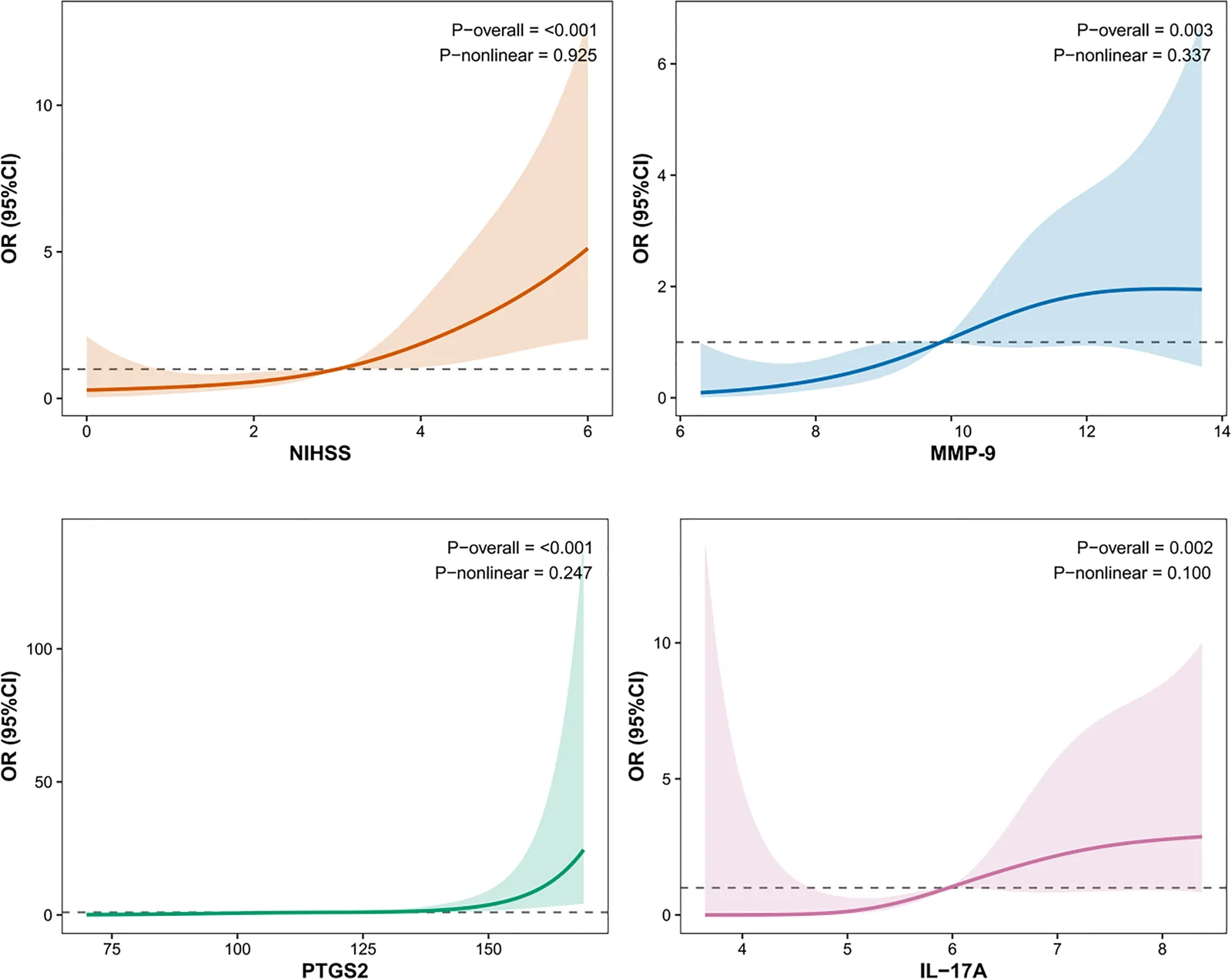
**Restricted cubic spline analysis of admission NIHSS score, MMP-9, PTGS2, and IL-17A in relation to poor 90-day outcome.** The plots show the adjusted associations between each continuous predictor and the odds of poor outcome, defined as a 90-day modified Rankin Scale score ≥ 3. Solid curves represent adjusted ORs, shaded areas indicate 95% CIs, and dashed horizontal lines indicate OR = 1. The models were adjusted for age, sex, hypertension, diabetes mellitus, coronary heart disease, and onset-to-blood sampling time. P-overall indicates the overall association between each predictor and poor outcome, whereas P-nonlinear indicates the test for deviation from linearity. All four predictors showed significant overall associations with poor outcome, with no statistically significant evidence of nonlinearity. **Abbreviations:** CI, confidence interval; IL-17A, interleukin-17A; MMP-9, matrix metalloproteinase-9; NIHSS, National Institutes of Health Stroke Scale; OR, odds ratio; PTGS2, prostaglandin-endoperoxide synthase 2.

**Figure 3.**
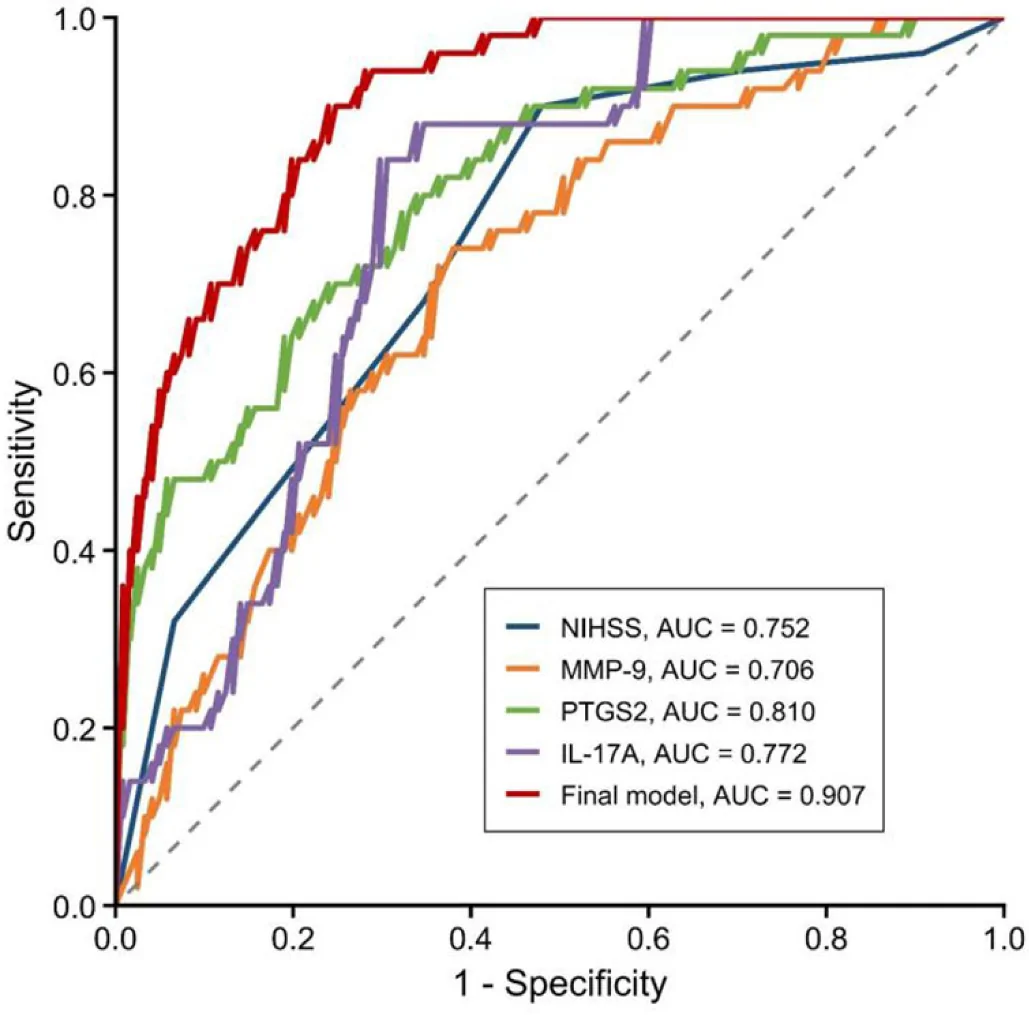
**Receiver operating characteristic curves for individual predictors and the final combined model in predicting poor 90-day outcome.** Receiver operating characteristic curves compare the discriminative performance of admission NIHSS score, MMP-9, PTGS2, IL-17A, and the final multivariable model for predicting poor 90-day functional outcome, defined as a modified Rankin Scale score ≥ 3. The final model, incorporating admission NIHSS score, MMP-9, PTGS2, and IL-17A, showed the highest discriminative ability, with an AUC of 0.907, compared with NIHSS alone (AUC = 0.752), MMP-9 (AUC = 0.706), PTGS2 (AUC = 0.810), and IL-17A (AUC = 0.772). The diagonal dashed line represents the line of no discrimination. **Abbreviations:** AUC, area under the receiver operating characteristic curve; IL-17A, interleukin-17A; MMP-9, matrix metalloproteinase-9; mRS, modified Rankin Scale; NIHSS, National Institutes of Health Stroke Scale; PTGS2, prostaglandin-endoperoxide synthase 2; ROC, receiver operating characteristic.

**Table 3:** Multivariable logistic regression analysis of independent predictors of adverse outcomes in elderly patients with AIS

**Variables**	**β**	**SE**	**Wald Z**	**OR (95% CI)**	***P* value**
Age	0.03	0.05	0.76	1.03 (0.95, 1.13)	0.449
Sex	0.74	0.55	1.36	2.11 (0.74, 6.45)	0.175
Hypertension	–0.32	0.56	−0.57	0.73 (0.24, 2.17)	0.566
Diabetes mellitus	0.05	0.56	0.09	1.05 (0.34, 3.15)	0.932
Coronary heart disease	0.01	0.84	0.01	1.01 (0.19, 5.27)	0.989
Onset-to-blood sampling time	0.04	0.05	0.79	1.04 (0.95, 1.14)	0.428
Admission NIHSS score	0.57	0.15	3.70	1.77 (1.34, 2.46)	<0.001
MMP-9	0.29	0.12	2.34	1.33 (1.06, 1.72)	0.020
PTGS2	0.04	0.01	3.60	1.04 (1.02, 1.06)	<0.001
IL-17A	0.58	0.21	2.82	1.79 (1.22, 2.74)	0.005

Multivariable logistic regression analysis was conducted to assess the independent associations between each variable and adverse outcomes, with results reported as ORs and 95% CIs. After adjusting for age, sex, hypertension, diabetes mellitus, coronary heart disease, and the time from onset to blood sampling, the admission NIHSS score, MMP-9, PTGS2, and IL-17A exhibited independent associations with poor outcomes. A *P* value of less than 0.05 was deemed statistically significant. **Abbreviations:** AIS, acute ischemic stroke; CI, confidence interval; IL-17A, interleukin-17A; MMP-9, matrix metalloproteinase-9; NIHSS, National Institutes of Health Stroke Scale; OR, odds ratio; PTGS2, prostaglandin-endoperoxide synthase 2; SE, standard error; β, beta coefficient.

### Construction of the final simplified prediction model

To enhance model parsimony and minimize the risk of overfitting, a final simplified prediction model was constructed, incorporating only the admission NIHSS score, MMP-9, PTGS2, and IL-17A as independent predictors. With 50 poor outcome events and four predictor parameters in the final model, the events-per-variable ratio was 12.5. Prior to model development, collinearity diagnostics were conducted, revealing that the variance inflation factor for each variable was less than 5, indicating no significant multicollinearity. Restricted cubic spline analysis was applied to these four continuous variables, demonstrating that the admission NIHSS score, MMP-9, PTGS2, and IL-17A were significantly associated with poor outcomes overall. Additionally, tests for nonlinearity were not statistically significant, suggesting a linear relationship with the outcome (Figure 2) and supporting their inclusion in the model as linear terms. The regression coefficients and OR values of the final simplified prediction model are presented in Table 4, which was subsequently utilized for nomogram construction and performance evaluation. It is important to note that Table 4 reflects a simplified model with only the four independent predictors, resulting in regression coefficients that differ from those in the screening model displayed in Table 3, which was adjusted for covariates.

**Table 4:** Regression coefficients and ORs of the final predictive model

**Variables**	**β**	**SE**	**Wald Z**	**OR (95% CI)**	***P* value**
(Intercept)	–13.83	2.28	−6.07	-	<0.001
Admission NIHSS score	0.52	0.14	3.66	1.69 (1.31, 2.30)	<0.001
MMP-9	0.33	0.12	2.73	1.39 (1.11, 1.78)	0.006
PTGS2	0.04	0.01	3.81	1.04 (1.02, 1.06)	<0.001
IL-17A	0.48	0.19	2.58	1.62 (1.14, 2.40)	0.010

The dependent variable in this study was poor outcome. The independent variables included the admission NIHSS score, MMP-9, PTGS2, and IL-17A, which were analyzed using a multivariable logistic regression model. The ORs and 95% CIs for the intercept term were not reported. The predicted probability of a poor outcome was calculated using the formula P: 1 / (1 + exp(−LP)), where LP represents the linear predictor defined as: LP: −13.83 + 0.52 × NIHSS + 0.33 × MMP-9 + 0.04 × PTGS2 + 0.48 × IL-17A. **Abbreviations:** CI, confidence interval; IL-17A, interleukin-17A; LP, linear predictor; MMP-9, matrix metalloproteinase-9; NIHSS, National Institutes of Health Stroke Scale; OR, odds ratio; PTGS2, prostaglandin-endoperoxide synthase 2; SE, standard error; β, beta coefficient.

### ROC analysis of individual biomarkers and combined models

ROC analysis indicated that the combined model achieved an AUC of 0.907 (95% CI: 0.864–0.950), compared to 0.752 for the NIHSS alone, 0.706 for MMP-9, 0.810 for PTGS2, and 0.772 for IL-17A (Figure 3). The absolute AUC improvement over the NIHSS alone was 0.155. Pairwise DeLong comparisons and multiplicity-adjusted *P* values are provided in Table S2. However, due to the multiple ROC comparisons performed, these results should be interpreted with caution and considered exploratory. The magnitude of the AUC differences suggests a notable improvement in discrimination of the combined model relative to individual predictors within the current cohort. Nonetheless, AUC-based improvement alone does not confirm clinically meaningful benefits, and the incremental predictive value of the biomarkers necessitates further validation through external studies and assessments of clinical utility beyond discrimination metrics.

### Model performance evaluation

The final simplified prediction model exhibited good discrimination, with an AUC of 0.907 (95% CI: 0.864–0.950) (Figure 4). At the optimal predicted probability cutoff of 0.284, determined by the maximum Youden index, the final multivariable model demonstrated a sensitivity of 92.0%, specificity of 74.4%, positive predictive value of 59.7%, negative predictive value of 95.7%, and overall classification accuracy of 79.5% for predicting poor outcomes at 90 days. A nomogram constructed from the final simplified prediction model illustrated that the admission NIHSS score, MMP-9, PTGS2, and IL-17A each contributed to the risk of poor outcomes to varying degrees; a higher total score correlated with an increased risk of poor outcomes (Figure 5). Following bootstrap correction, the C-index was 0.897, indicating good model stability. Calibration assessments revealed strong agreement between predicted and observed probabilities. The bootstrap-corrected calibration intercept was 0.0004, indicating minimal systematic over- or underestimation of risk, while the bootstrap-corrected calibration slope was 0.916, suggesting only mild overfitting. The Hosmer–Lemeshow test did not indicate a significant lack of fit (*P* ═ 0.928), and the bootstrap calibration curve exhibited good visual concordance between predicted and observed probabilities (Figure 6). DCA demonstrated a higher net benefit for the model compared to the treat-all and treat-none strategies across a range of threshold probabilities, particularly within the clinically relevant range of approximately 10%–50% (Figure 7). Lower thresholds, around 10%–20%, may suggest relatively low-risk actions such as closer neurological monitoring, more frequent reassessment, early complication prevention, and early rehabilitation evaluation. Moderate thresholds, approximately 20%–50%, may imply more selective escalation of care, including intensified monitoring, structured complication prevention, enhanced communication with patients and families, and more intensive follow-up planning. However, DCA does not establish that biomarker-guided management improves outcomes; it merely indicates potential net benefits under specified threshold assumptions. Given that model development and evaluation were conducted within the same modest single-center cohort, the discrimination, calibration, and net-benefit estimates may still be optimistic despite bootstrap correction. Therefore, these performance results should be regarded as preliminary internal estimates, and the model should not be considered clinically applicable until validated externally in larger and more heterogeneous cohorts.

**Figure 4.**
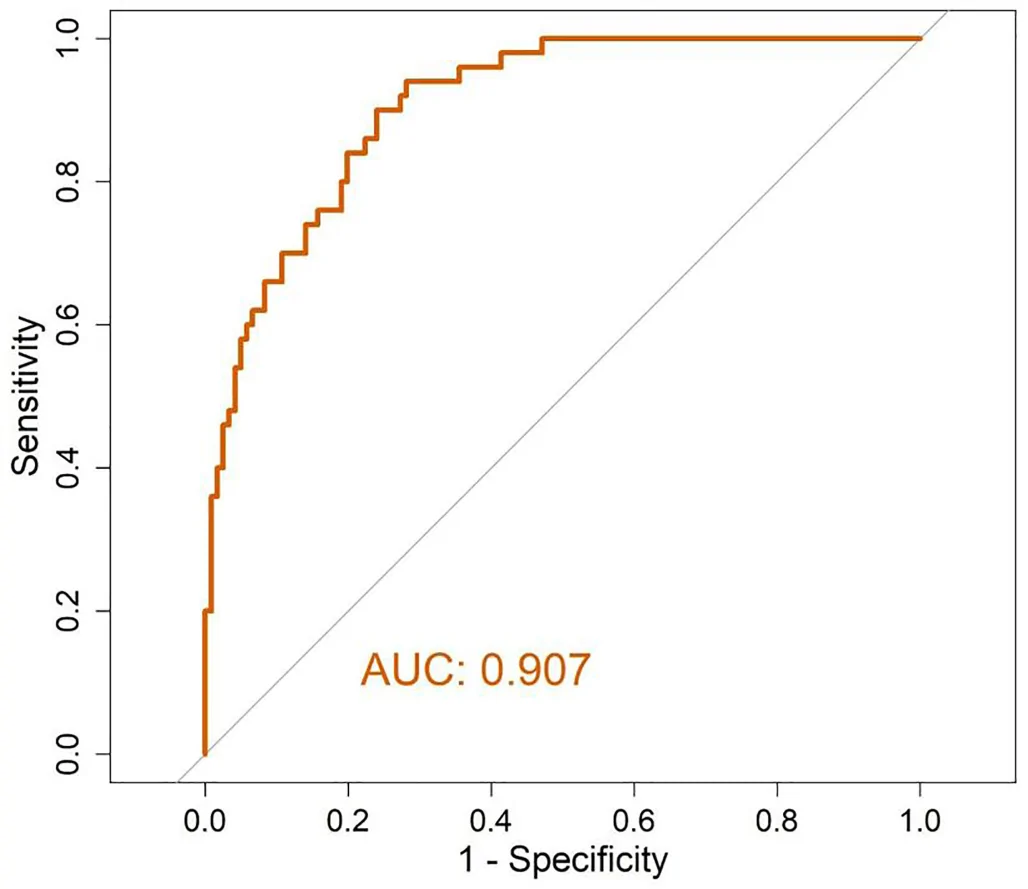
**Receiver operating characteristic curve of the final prediction model for poor 90-day outcome.** The receiver operating characteristic curve shows the discriminative performance of the final multivariable logistic regression model for predicting poor 90-day functional outcome in older patients with acute ischemic stroke. The model incorporated admission NIHSS score, MMP-9, PTGS2, and IL-17A. Poor outcome was defined as a 90-day modified Rankin Scale score ≥ 3. The model demonstrated good discrimination, with an AUC of 0.907. The diagonal reference line indicates no discriminative ability. **Abbreviations:** AIS, acute ischemic stroke; AUC, area under the receiver operating characteristic curve; IL-17A, interleukin-17A; MMP-9, matrix metalloproteinase-9; mRS, modified Rankin Scale; NIHSS, National Institutes of Health Stroke Scale; PTGS2, prostaglandin-endoperoxide synthase 2; ROC, receiver operating characteristic.

**Figure 5.**
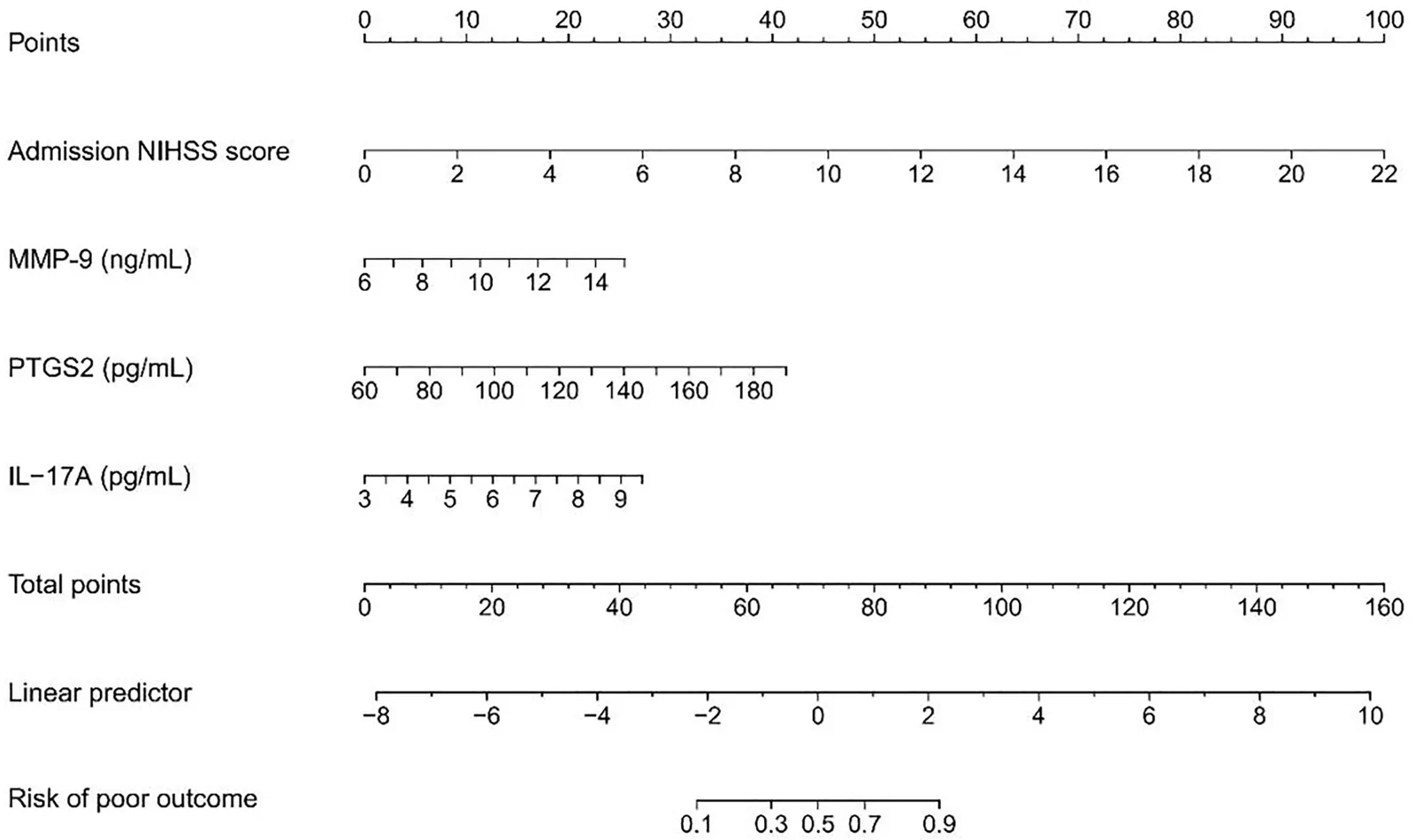
**Nomogram for predicting poor 90-day outcome in older patients with acute ischemic stroke.** The nomogram was derived from the final simplified multivariable logistic regression model and incorporates admission NIHSS score, MMP-9, PTGS2, and IL-17A. For each predictor, a vertical line is drawn upward to the “Points” scale to determine the corresponding score; the individual scores are then summed to obtain the “Total points,” which correspond to the linear predictor and the estimated probability of poor outcome, defined as a 90-day modified Rankin Scale score ≥ 3. Higher total points indicate a higher predicted risk of poor outcome. **Abbreviations:** IL-17A, interleukin-17A; MMP-9, matrix metalloproteinase-9; mRS, modified Rankin Scale; NIHSS, National Institutes of Health Stroke Scale; PTGS2, prostaglandin-endoperoxide synthase 2.

**Figure 6.**
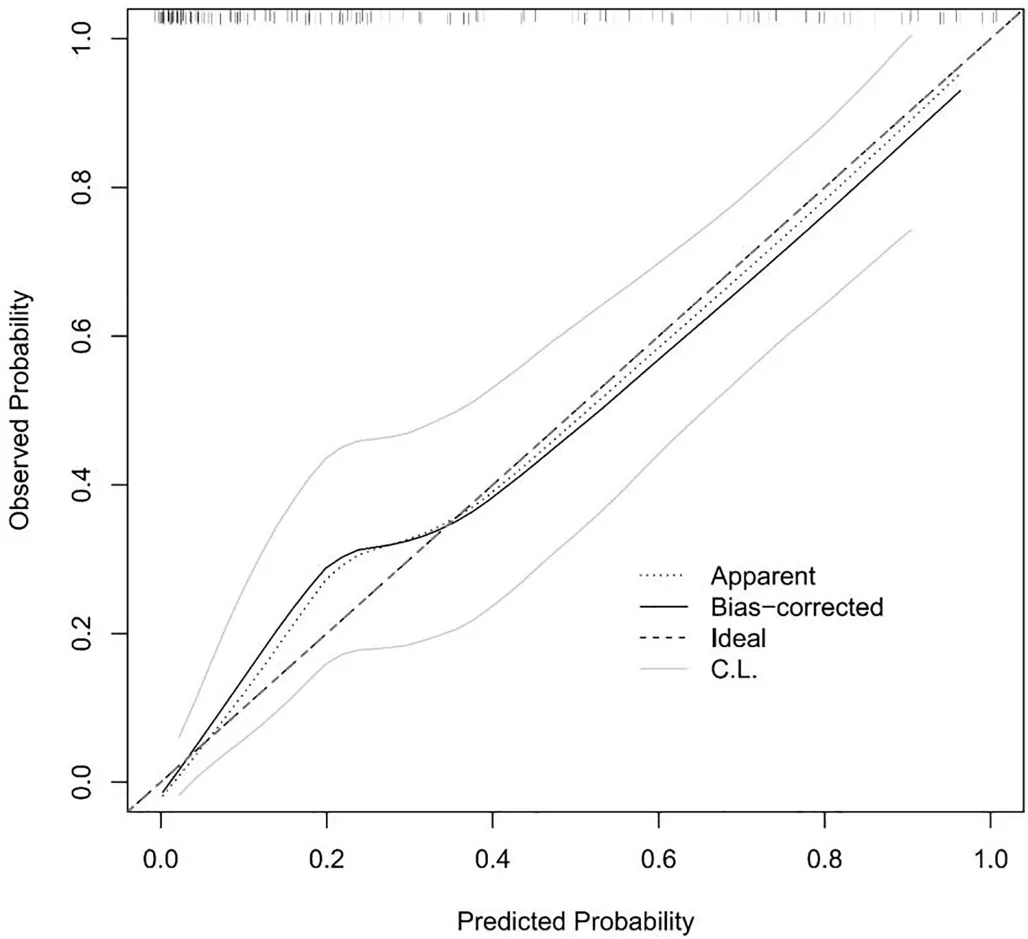
**Calibration plot of the final prediction model for poor 90-day outcome.** The calibration plot compares the predicted and observed probabilities of poor 90-day outcome in older patients with AIS, defined as a mRS score ≥ 3. The final model incorporated admission NIHSS score, MMP-9, PTGS2, and IL-17A. The dashed diagonal line represents ideal calibration, the dotted line represents apparent calibration, and the solid line represents bootstrap bias-corrected calibration after internal validation. The light gray curves indicate confidence limits. Closer agreement between the bias-corrected curve and the ideal line indicates better agreement between predicted and observed risks. **Abbreviations:** AIS, acute ischemic stroke; IL-17A, interleukin-17A; MMP-9, matrix metalloproteinase-9; mRS, modified Rankin Scale; NIHSS, National Institutes of Health Stroke Scale; PTGS2, prostaglandin-endoperoxide synthase 2.

**Figure 7.**
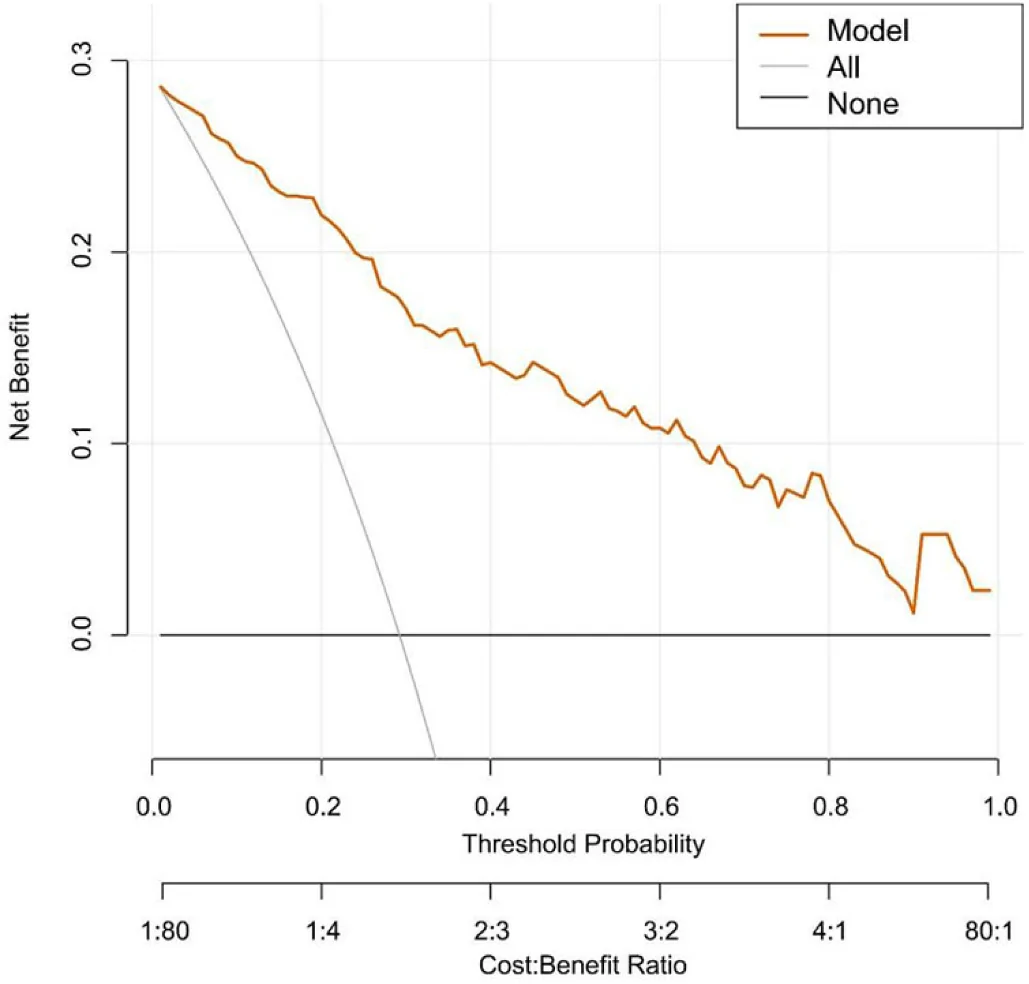
**DCA of the final prediction model for poor 90-day outcome.** DCA was used to evaluate the potential clinical utility of the final model for predicting poor 90-day functional outcome in older patients with AIS, defined as an mRS score ≥ 3. The model incorporated admission NIHSS score, MMP-9, PTGS2, and IL-17A. The x-axis shows the threshold probability, the lower axis shows the corresponding cost:benefit ratio, and the y-axis shows net benefit. The “All” strategy assumes that all patients would receive intensified monitoring, risk management, or follow-up planning, whereas the “None” strategy assumes that no patients would receive additional measures. The model showed a higher net benefit than both reference strategies across a broad range of threshold probabilities, particularly within the clinically plausible exploratory range of approximately 10%–50%. These findings suggest potential usefulness for early risk stratification but do not establish that biomarker-guided management improves outcomes. **Abbreviations:** AIS, acute ischemic stroke; DCA, decision curve analysis; IL-17A, interleukin-17A; MMP-9, matrix metalloproteinase-9; mRS, modified Rankin Scale; NIHSS, National Institutes of Health Stroke Scale; PTGS2, prostaglandin-endoperoxide synthase 2.

## Discussion

In this study, we developed and internally validated a simplified model for predicting 90-day poor outcomes in older patients with AIS by integrating the admission NIHSS score with inflammation-related serum biomarkers. Admission NIHSS score, MMP-9, PTGS2, and IL-17A were independently associated with poor outcomes, and the combined model demonstrated strong apparent discrimination and acceptable internal calibration. In contrast to most previous studies, which included AIS populations of mixed ages, our research specifically focused on the older subgroup, characterized by greater clinical complexity. The rationale for this model lies in the fact that NIHSS reflects the severity of neurological deficits, while MMP-9, PTGS2, and IL-17A may provide complementary biological insights related to blood–brain barrier injury, extracellular matrix remodeling, prostaglandin-mediated inflammation, and immune activation. Unlike many existing stroke prognostic scores that primarily rely on clinical and conventional laboratory variables, our model explores the potential of inflammation-related biomarkers to offer additional prognostic information beyond admission NIHSS in older patients with AIS. However, since no formal comparison with existing models was conducted, the current findings should not be interpreted as evidence of superiority, and external validation remains necessary. The model’s development and evaluation occurred within the same modest single-center cohort, which included only 50 poor outcome events, potentially inflating performance estimates despite bootstrap internal validation. While bootstrap correction can assess internal optimism, it cannot replace external validation in independent populations. Additionally, although the final model was parsimonious, it was derived following an adjusted evaluation step that included prespecified covariates, which may also have introduced selection-related optimism. Furthermore, these biomarkers are not routinely tested at the bedside in many hospitals, limiting immediate clinical implementation. Consequently, the present model should be viewed as an exploratory, hypothesis-generating biomarker-based framework rather than a clinically applicable prediction tool at this stage.

The mild case mix of the present cohort is significant for interpretation. The median NIHSS score was 2 in the favorable outcome group and 4 in the poor outcome group, with large infarctions being rare. This indicates that the cohort is primarily composed of patients with mild-to-moderate AIS rather than representing the full spectrum of stroke severity. This may be partially attributed to the exclusion of patients receiving intravenous thrombolysis or endovascular treatment. In older adults with mild AIS, the 90-day functional outcome may be influenced not only by the acute neurological deficit but also by factors such as frailty, comorbidities, cognitive reserve, premorbid functional status, and existing cerebrovascular burden. Consequently, the lack of significant associations between infarct size and location may reflect limited variability in infarct burden rather than an absence of prognostic relevance. The generalizability of the model to patients with moderate-to-severe AIS, large-vessel occlusion, large territorial infarction, or those undergoing reperfusion therapy remains uncertain. Furthermore, the exclusion of patients receiving thrombolysis or endovascular treatment may have altered the distribution of inflammation-related biomarkers, as these patients often present with different stroke severities, infarct burdens, treatment responses, and blood-brain barrier injury profiles. Therefore, the observed model performance in this conservatively treated cohort should not be directly extrapolated to populations undergoing reperfusion therapy.

The associations of MMP-9, IL-17A, and PTGS2 with poor outcomes are biologically plausible. MMP-9 can degrade vascular basement membrane components and disrupt blood-brain barrier integrity, and elevated MMP-9 levels have been associated with hemorrhagic transformation, early neurological deterioration, and poor outcomes following AIS [19–22]. IL-17A, an effector cytokine involved in Th17-related inflammation, can promote microglial activation, chemokine expression, neutrophil infiltration, and secondary brain injury [23–25], and has also been linked to post-stroke cognitive decline [26]. PTGS2 is rapidly induced following cerebral ischemia, participating in prostaglandin-mediated inflammation, oxidative stress, and potentially ferroptosis-related injury [13, 14, 27, 28]. In older adults, immunosenescence, chronic low-grade inflammation, and age-related blood-brain barrier vulnerability may further amplify these pathways [29–32]. While these mechanisms support the potential relevance of these biomarkers, causal relationships cannot be inferred from the current observational study.

The incremental value of adding inflammatory biomarkers to the NIHSS should be interpreted with caution. Although the combined model demonstrated a higher AUC than the NIHSS alone, an improved AUC does not necessarily equate to clinically meaningful benefits. Given that NIHSS scores were relatively low and narrowly distributed in this cohort, the apparent contribution of biomarkers may have been overstated. DCA suggested potential net benefits within clinically plausible threshold ranges; however, this analysis was exploratory and evaluated internally only. If externally validated, biomarker-enhanced risk stratification could aid in identifying older patients with seemingly mild deficits who remain at increased risk and may require closer monitoring, early rehabilitation planning, complication prevention, or structured follow-up. Nonetheless, this study did not assess whether biomarker-informed decisions would alter management or improve outcomes. Future research should evaluate the incremental value beyond AUC through external validation, calibration, risk reclassification metrics, clinical impact curves, and prospective assessments of clinical utility.

Other sources of outcome heterogeneity should be considered in subsequent studies. Pre-existing white matter alterations, cerebral small vessel disease, cognitive impairment, metabolic vascular burden, and stroke subtype heterogeneity may influence recovery after AIS, particularly in older adults. Previous studies have linked white matter abnormalities and lacunar stroke subtypes to cognitive impairment, vascular risk profiles, and outcome heterogeneity following stroke [33, 34]. Therefore, future investigations should incorporate detailed neuroimaging, cognitive and metabolic assessments, and perform stratified analyses by stroke subtype, particularly distinguishing between lacunar and non-lacunar stroke.

This study has several limitations. First, it was a single-center prospective observational study with a modest sample size and only 50 poor outcome events. Although the final model included four predictors and bootstrap resampling was employed for internal validation, the model was developed and evaluated within the same cohort. Furthermore, the final simplified model was derived after assessing candidate predictors in an adjusted multivariable model that included prespecified covariates, potentially contributing to selection-related optimism. Therefore, optimism in discrimination, calibration, and decision-curve performance cannot be excluded, and the model should be regarded as exploratory and hypothesis-generating until externally validated. Second, the cohort was selectively composed and relatively mild. Patients receiving intravenous thrombolysis or endovascular treatment were excluded, admission NIHSS scores were overall low, and large infarctions were uncommon. This selection bias may have shifted the distributions of PTGS2, MMP-9, and IL-17A, as biomarker levels may vary according to stroke severity, infarct burden, reperfusion status, and treatment-related inflammatory responses. In older adults with mild AIS, the 90-day functional outcome may also be influenced by frailty, comorbidities, cognitive reserve, premorbid functional status, and pre-existing cerebrovascular disease, which were not comprehensively assessed. These factors limit the generalizability of the findings to patients with more severe strokes, large-vessel occlusions, large territorial infarctions, or those undergoing reperfusion therapy. Third, PTGS2, MMP-9, and IL-17A are not standard bedside tests in many clinical settings and were measured only once within 24 h after stroke onset. Their clinical implementation would necessitate standardized assays, validated reference ranges, acceptable turnaround times, and cost-effectiveness evaluations. Fourth, this study did not compare the proposed model with established stroke prognostic scores, formally assess risk reclassification or clinical impact curves, or determine whether biomarker-guided risk stratification would alter management or improve outcomes. Fifth, due to the limited sample size, stratified analyses by TOAST subtype or by lacunar vs non-lacunar stroke were not performed. Finally, white matter lesion burden, metabolic syndrome, and cognitive impairment were not systematically evaluated. Future multicenter studies with larger and more heterogeneous cohorts, serial biomarker measurements, detailed neuroimaging, and standardized cognitive assessments are warranted.

## Conclusion

In this selected, relatively mild, conservatively treated older AIS cohort, the model incorporating admission NIHSS score, PTGS2, MMP-9, and IL-17A demonstrated good apparent discrimination and internal calibration. However, due to the modest sample size and the development and evaluation of the model being conducted within the same dataset, its performance and the apparent incremental value of biomarkers may be optimistic despite bootstrap correction. Additionally, the biomarker panel is not routinely available in many clinical settings, and the exclusion of patients receiving thrombolysis or endovascular treatment limits generalizability. Therefore, this model should be considered exploratory and hypothesis-generating rather than clinically applicable at this stage. External validation in larger, multicenter, independent, and more heterogeneous cohorts, including patients with more severe strokes and those receiving reperfusion therapy, is essential before clinical application can be contemplated.

## Supplemental data

**Table S1:** Logistic regression results for biomarkers per one standard deviation increase

**Variable**	**Level**	**Univariable OR (95% CI)**	**Univariable *P* value**	**Multivariable OR (95% CI)**	**Multivariable *P* value**
MMP-9	per 1 SD increase	2.18 (1.52–3.21)	<0.001	1.66 (1.05–2.68)	0.033
PTGS2	per 1 SD increase	4.15 (2.63–7.05)	<0.001	3.03 (1.83–5.37)	<0.001
IL-17A	per 1 SD increase	2.99 (1.99–4.71)	<0.001	2.36 (1.46–4.03)	<0.001

Biomarker variables were standardized as z-scores, with odds ratios reported for each 1 standard deviation increase. The multivariable model was adjusted for age, sex, hypertension, diabetes mellitus, coronary heart disease, and the duration from onset to blood sampling. **Abbreviations:** CI, confidence interval; IL-17A, interleukin-17A; MMP-9, matrix metalloproteinase-9; OR, odds ratio; PTGS2, prostaglandin-endoperoxide synthase 2; SD, standard deviation.

**Table S2:** Pairwise comparisons of AUCs between the combined model and individual predictors

**Comparison**	**Model 1**	**AUC 1**	**Model 2**	**AUC 2**	**Absolute AUC difference**	**DeLong *P* value**	**Bonferroni-adjusted *P* value**	**FDR-adjusted *P* value**
Combined model vs NIHSS	Combined model	0.907	NIHSS	0.752	0.155	<0.001	<0.001	<0.001
Combined model vs MMP-9	Combined model	0.907	MMP-9	0.706	0.201	<0.001	<0.001	<0.001
Combined model vs PTGS2	Combined model	0.907	PTGS2	0.810	0.097	<0.001	0.003	<0.001
Combined model vs IL-17A	Combined model	0.907	IL-17A	0.772	0.135	<0.001	<0.001	<0.001

The combined model represents the final nomogram incorporating the admission NIHSS score, MMP-9, PTGS2, and IL-17A. The AUC values were compared using the DeLong test. Due to the multiple pairwise ROC comparisons conducted, nominal *P* values should be interpreted with caution. Bonferroni- and FDR-adjusted *P* values are also provided. It is essential to prioritize the magnitude of the absolute AUC differences rather than focusing solely on statistical significance. **Abbreviations:** AUC, area under the receiver operating characteristic curve; FDR, false discovery rate; IL-17A, interleukin-17A; MMP-9, matrix metalloproteinase-9; NIHSS, National Institutes of Health Stroke Scale; PTGS2, prostaglandin-endoperoxide synthase 2; ROC, receiver operating characteristic.

## Data Availability

The original contributions presented in the study are included in the article. Further inquiries can be directed to the corresponding author.

## References

[1] Feigin VL, Stark BA, Johnson CO, Roth GA, Bisignano C, Abady GG (2021). Global, regional, and national burden of stroke and its risk factors, 1990–2019:a systematic analysis for the Global Burden of Disease Study 2019. Lancet Neurol.

[2] Zhu W, He X, Huang D, Jiang Y, Hong W, Ke S (2025). Global and regional burden of ischemic stroke disease from 1990 to 2021:an age-period-cohort analysis. Transl Stroke Res.

[3] Gasull T, Arboix A (2022). Molecular mechanisms and pathophysiology of acute stroke: emphasis on biomarkers in the different stroke subtypes. Int J Mol Sci.

[4] Knoflach M, Matosevic B, Rücker M, Furtner M, Mair A, Wille G (2012). Functional recovery after ischemic stroke: a matter of age; data from the Austrian Stroke Unit Registry. Neurology.

[5] Miranda LA, Luvizutto GJ, Bessornia PAC, Furlan NE, Winckler FC, Ferreira NC (2024). To what extent does frailty mediate the association between age and the outcomes of brain reperfusion following acute ischemic stroke?. Front Aging Neurosci.

[6] Drozdowska BA, Singh S, Quinn TJ (2019). Thinking about the future: a review of prognostic scales used in acute stroke. Front Neurol.

[7] Simats A, Liesz A (2022). Systemic inflammation after stroke: implications for post-stroke comorbidities. EMBO Mol Med.

[8] Zietz A, Gorey S, Kelly PJ, Katan M, McCabe JJ (2024). Targeting inflammation to reduce recurrent stroke. Int J Stroke.

[9] Franceschi C, Garagnani P, Parini P, Giuliani C, Santoro A (2018). Inflammaging: a new immune-metabolic viewpoint for age-related diseases. Nat Rev Endocrinol.

[10] Iordache MP, Buliman A, Costea-Firan C, Gligore TCI, Cazacu IS, Stoian M (2025). Immunological and inflammatory biomarkers in the prognosis, prevention, and treatment of ischemic stroke: a review of a decade of advancement. Int J Mol Sci.

[11] Turner RJ, Sharp FR (2016). Implications of MMP9 for blood–brain barrier disruption and hemorrhagic transformation following ischemic stroke. Front Cell Neurosci.

[12] Wang J, Gao Y, Yuan Y, Wang H, Wang Z, Zhang X (2024). Th17 cells and IL-17A in ischemic stroke. Mol Neurobiol.

[13] Iadecola C, Gorelick PB (2005). The Janus face of cyclooxygenase-2 in ischemic stroke: shifting toward downstream targets. Stroke.

[14] Iadecola C (2003). Cyclooxygenase-2 and stroke: the long and short of it. Ann Neurol.

[15] Liu L, Li Z, Zhou H, Duan W, Huo X, Xu W (2023). Chinese Stroke Association guidelines for clinical management of ischaemic cerebrovascular diseases: executive summary and 2023 update. Stroke Vasc Neurol.

[16] Brott T, Adams HP Jr, Olinger CP, Marler JR, Barsan WG, Biller J (1989). Measurements of acute cerebral infarction: a clinical examination scale. Stroke.

[17] Adams HP Jr, Bendixen BH, Kappelle LJ, Biller J, Love BB, Gordon DL (1993). Classification of subtype of acute ischemic stroke: definitions for use in a multicenter clinical trial; TOAST. Trial of Org 10172 in Acute Stroke Treatment. Stroke.

[18] van Swieten JC, Koudstaal PJ, Visser MC, Schouten HJ, van Gijn J (1988). Interobserver agreement for the assessment of handicap in stroke patients. Stroke.

[19] Zheng X, Zhong C, Zhu Z, Zhang K, Peng H, Xu T (2021). Association between serum matrix metalloproteinase-9 and poor prognosis in acute ischemic stroke patients: the role of dyslipidemia. Nutr Metab Cardiovasc Dis.

[20] Zhao BQ, Wang S, Kim HY, Storrie H, Rosen BR, Mooney DJ (2006). Role of matrix metalloproteinases in delayed cortical responses after stroke. Nat Med.

[21] Castellanos M, Leira R, Serena J, Pumar JM, Lizasoain I, Castillo J (2003). Plasma metalloproteinase-9 concentration predicts hemorrhagic transformation in acute ischemic stroke. Stroke.

[22] Zhong C, Yang J, Xu T, Xu T, Peng Y, Wang A (2017). Serum matrix metalloproteinase-9 levels and prognosis of acute ischemic stroke. Neurology.

[23] Chen X, Zhang Y, Ding Q, He Y, Li H (2023). Role of IL-17A in different stages of ischemic stroke. Int Immunopharmacol.

[24] Zhang Q, Liao Y, Liu Z, Dai Y, Li Y, Li Y (2021). Interleukin-17 and ischaemic stroke. Immunology.

[25] Gelderblom M, Weymar A, Bernreuther C, Velden J, Arunachalam P, Steinbach K (2012). Neutralization of the IL-17 axis diminishes neutrophil invasion and protects from ischemic stroke. Blood.

[26] Lu T, Ma L, Xu Q, Wang X (2022). Blood Th17 cells and IL-17A as candidate biomarkers estimating the progression of cognitive impairment in stroke patients. J Clin Lab Anal.

[27] Nakayama M, Uchimura K, Zhu RL, Nagayama T, Rose ME, Stetler RA (1998). Cyclooxygenase-2 inhibition prevents delayed death of CA1 hippocampal neurons following global ischemia. Proc Natl Acad Sci U S A.

[28] Xu Y, Liu Y, Li K, Yuan D, Yang S, Zhou L (2022). COX-2/PGE2 pathway inhibits the ferroptosis induced by cerebral ischemia reperfusion. Mol Neurobiol.

[29] Erdő F, Denes L, de Lange E (2017). Age-associated physiological and pathological changes at the blood–brain barrier: a review. J Cereb Blood Flow Metab.

[30] Qi L, Wang F, Sun X, Li H, Zhang K, Li J (2024). Recent advances in tissue repair of the blood–brain barrier after stroke. J Tissue Eng.

[31] Cao Y, Xu W, Liu Q (2024). Alterations of the blood–brain barrier during aging. J Cereb Blood Flow Metab.

[32] Li Q, Ding S, Wang YM, Xu X, Shen Z, Fu R (2017). Age-associated alteration in Th17 cell response is related to endothelial cell senescence and atherosclerotic cerebral infarction. Am J Transl Res.

[33] Segura B, Jurado MA, Freixenet N, Bargalló N, Junqué C, Arboix A (2010). White matter fractional anisotropy is related to processing speed in metabolic syndrome patients: a case-control study. BMC Neurol.

[34] Arboix A, Massons J, García-Eroles L, Targa C, Comes E, Parra O (2010). Nineteen-year trends in risk factors, clinical characteristics and prognosis in lacunar infarcts. Neuroepidemiology.

